# Altered Brain Structure in the Patients with Painful Temporomandibular Disorders: A Pilot Surface-based Morphometry

**DOI:** 10.2174/0115734056355184250307055847

**Published:** 2025-04-29

**Authors:** Xin Li, YuJiao Jiang, Zhiye Chen

**Affiliations:** 1 Department of Radiology, Hainan Hospital of PLA General Hospital, Sanya 572013, China; 2 Department of Nuclear Medicine, Xinqiao Hospital, Army Medical University, Chongqing 400037, China; 3 School of Medical Imaging, Bengbu Medical University, Bengbu 233030, China

**Keywords:** Temporomandibular disorders, Pain, Brain structure, Surface-based morphometry, Normal control, Voxel-based morphometry

## Abstract

**Background:**

Pain is a significant indicator of temporomandibular disorders (TMDs), which are impacted by a complex process. Recently, the evolution and chronification of painful TMD (p-TMD) have been facilitated by central nervous system mechanisms. Therefore, the purpose of this study was to investigate the aberrant brain structure in p-TMD patients using surface-based morphometry (SBM) analysis.

**Methods:**

This study recruited forty-one p-TMD patients and 33 normal controls (NC) who underwent high-resolution brain structural imaging on a 3.0T MR scanner. SBM analysis was applied to the brain structural images, and the surface parameters, including the cortical thickness, fractal dimension, sulcus depth, and gyrification index, were measured. The independent two-sample t-test by SPM12, with age and gender as covariates, was used to investigate the differences in p-TMD patients compared with the NC.

**Results:**

The p-TMD group had significantly decreased cortical thickness in the left lateral occipital cortex and significantly decreased fractal dimension in the left paracentral, right pars opercularis, right rostral middle frontal, left lingual, and right inferior temporal cortices when compared with NCs. However, there were no significant differences in sulcal depth and gyrification index between the two groups.

**Conclusion:**

This study demonstrated decreased cortical thickness and fractal dimension in p-TMD patients, which may be associated with abnormal neural mechanisms underlying the brain's processing of emotions and pain. The SBM technology may offer additional independent morphological characteristics for investigating the structure of the brain.

## 1 BACKGROUND\INTRODUCTION

Up to 40% of the general population may experience chronic non-malignant pain, which has become a serious public health concern [1]. Temporomandibular disorders (TMDs), with an estimated prevalence of 31% in adults and 11% in children, are among the most common chronic pain problems [2]. Clinical indicators like temporomandibular joint sounds and impaired mandibular movement are primarily used to diagnose TMD discomfort [3]. Additionally, because it is thought to be a comprehensive illness that combines a number of harmful dental habits with detrimental psychological variables, it is crucial to investigate the pathophysiological process underlying painful TMD (p-TMD) [4].

It was believed that peripheral problems would cause TMD pain; however, in many p-TMD patients, no peripheral pain generator can be identified [5-7]. Numerous studies using neuroimaging techniques have looked into the functional and structural changes in the brain, providing evidence that the central nervous system may play a role in the evolution and chronification of p-TMD patients as well as in patients with other chronic pain conditions [8-10]. Additionally, a fresh viewpoint on the brain structural alterations in p-TMD patients may be obtained through brain morphological investigation. For measuring brain structure, surface-based morphometry (SBM) was thought to be superior to voxel-based morphometry (VBM). This is because the traditional VBM approach may depict changes in local brain volume based on voxel level, while brain volume may be influenced by several variables, including cortical thickness, surface area, and sulci depth. In the meanwhile, VBM relied on the alignment and image registration procedure, but some differences existed [11]. SBM, however, is able to provide sub-millimeter measurements of complex cortical topological structure, including cortical thickness and changed gyrification [12]. Therefore, it can serve as a supplement to the shortcomings of the single brain volume measurement of VBM and provide a more thorough reflection of the more nuanced structural properties of the cerebral surface cortex. In this study, we investigate the potential qualitative and quantitative changes in the brain cortical morphology in p-TMD patients using the SBM method, which may yield more independent morphological metrics.

The purpose of our study was to investigate the cortical surface characteristics changed in p-TMD subjects. There-fore,we progressively recruited 41 p-TMD patients and 33 normal controls (NC), used the SBM method to assess the cortical structural changes, and tried to offer new insights into the pathophysiological process of p-TMD.

## 2 METHODS

### 2.1 Subjects

Inclusion criteria for the p-TMD group were listed as follows: (1) TMD patients diagnosed by the Diagnostic Criteria for Temporomandibular Disorders [13, 14] with mainly persisted joint pain for more than 3 months during rest or function; (2) being in the interictal stage at least three days after a pain attack; (3) no other treatment, such as psycho-therapy and acupuncture administered that could potentially affect neurological function; (4) no accompanying pain in other parts of the body; (5) no history of neurological or psychiatric disorders or cognitive dysfunction; and (6) adults aged 18-60 and right-handed. Moreover, the sex-, age- and education-matched NCs should never have had any primary TMJ-related diseases or other types of pain in the past years.

The clinical pain experience of p-TMD patients was assessed using the Visual Analogue Scale (VAS). This study used the Axis II of DC/TMD to estimate the clinical information of all the subjects, including the Jaw Functional Limitation Scale (JFLS) from four dimensions (chewing, movement, communication, and general function) to evaluate the degree of mandibular function limitation; Oral Behavior Checklist (OBC) to assess the frequency of oral bad behavior; and Patient Health Questionnaire-9 (PHQ-9), Generalized Anxiety Disorder-7 (GAD-7) and Patient Health Questionnaire-15 (PHQ-15) for the evaluation of depressive, anxiety, and somatization symptoms separately. Additionally, the Hamilton Anxiety Scale (HAMA) and Hamilton Depression Scale (HAMD) were utilized for the psychology evaluation of all individuals.

All enrolled subjects provided written informed consent prior to the acquisition of MR images, and the study was approved by the ethics committee of the Hainan Hospital of PLA General Hospital (S2022-03), compliant with the ethical guidelines of the Declaration of Helsinki.

### 2.2 MRI Data Acquisition

All brain MR imaging data collection was performed on a 3.0T MRI scanner (Philips, Ingenia CX, Netherlands) using a 32-channel head receiving coil. For each participant, three-dimensional T1-weighted structural images were collected using a fast field echo sequence (TR = 6.6 ms, TE = 3.0 ms, thickness = 1mm, matrix size = 220 × 240, FOV = 22 × 24 cm^2^, voxel size = 1 × 1 × 1 mm^3^, number of slices = 150). All the participants should never have had MRI contraindications, including metal clips within the body and claustrophobia, and were instructed to avoid alcohol, nicotine, caffeine, and other substances intake at least 12 hours before MRI examinations. They were instructed to lie still in a relaxed position with their eyes closed while remaining awake and avoiding any specific thoughts. All structural MRI scans were visually inspected by the same technician for any significant brain abnormalities or head movements.

### 2.3 Data Processing

Surface-based morphometric analyses were performed using the MATLAB 2021b (The Mathworks, Natick, MA, USA) based Computational Anatomy Toolbox (CAT12, http://www.neuro.uni-jena.de/cat/), which is an extension of SPM12 (Wellcome Department of Cognitive Neurology, London, UK) (https://www.fil.ion.ucl.ac.uk/spm/).

The image pre-processing steps were as follows:

#### 2.3.1 Set origin

T1 imaging data were converted from DICOM into NIfTI, and all the raw image origin was set at anterior commissure (0, 0, 0).

#### 2.3.2 Imaging preprocessing

This included affine regularization, inhomogeneity correction, skull- stripping, and spatial registration.

#### 2.3.3 SBM image Processing

This included segmentation, topological correction, spherical mapping, and spherical registration.

#### 2.3.4 Surface Parameters Estimation

This included cortical thickness (CT), fractal dimension (FD), gyrification index (GI), and sulcal depth (SD).

#### 2.3.5 Smoothening

 A 15-mm full-width half maximum (FWHM) Gaussian kernel was used to smooth the resampled segmented data for CT, and 20-mm FWHM Gaussian kernel was used for FD, GI, and SD analysis, as per the default recommended pipeline of CAT12.

### 2.4 Statistical Analysis

An independent two-sample t-test by SPM12 was used for the voxel-based analysis, with age and gender as covariates, to investigate the differences in p-TMD patients compared with the NC. A *P* value of <0.001 without false discovery rate correction was considered to indicate a significant difference. The clinical data were processed by IBM-SPSS 25. The quantitative data with normal distribution were presented as mean ± SD and performed with an independent sample *t*-test. The quantitative data with non-normal distribution was presented by median (the first quartile, the third quartile), and performed with Chi-Square test.

## 3 RESULT

### 3.1 Demography and Clinical Characteristics

Tables **1** and **2 ** depict the demographic and clinical characteristics of the subjects involved in this study (41 p-TMD patients and 33 NCs). There were no significant differences in age, gender, GAD-7, PHQ-9, PHQ-15, OBC, HAMA, and HAMD scores between the two groups (*P*>0.05). p-TMD patients had a significantly higher JFLS score (35.00(50.00, 16.00)) than NC (3.00(6.50, 0.00)) (*P*<0.05). The VAS score in the p-TMD group was 3.00 (4.50-2.00).

### 3.2 The Brain Regions with Morphological Changes in the p-TMD Group

**Fig. (1):**
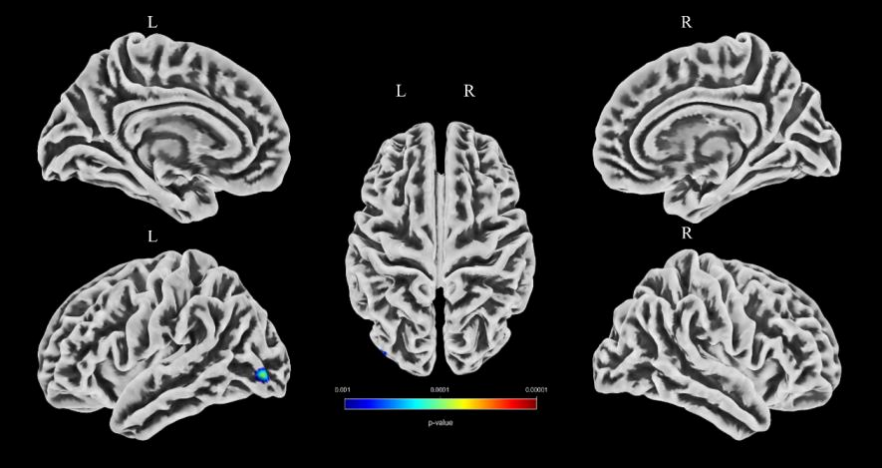
The brain region with a decreased cortical thickness over the whole brain in the p-TMD group.

**Fig. (2):**
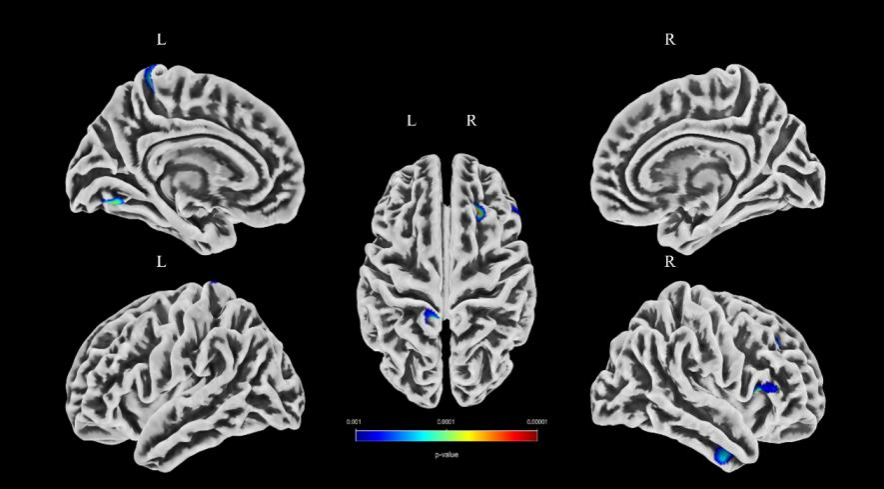
The brain regions with a decreased fractal dimension over the whole brain in the p-TMD group.

The brain region with decreased CT was in the left lateral occipital (Table **3** and Fig. **1**), while there were no thickening brain regions detected in p-TMD patients compared with NCs. The brain regions with decreased FD were included as follows (Table **4** and Fig. **2**): left paracentral, right pars opercularis, right rostral middle frontal, left lingual, and right inferior temporal. However, there were no brain regions with increased FD in p-TMD patients compared with NCs. Moreover, there were no significant differences in GI and SD between the two groups.

## 4 DISCUSSION

The current study used SBM analysis to conduct a preliminary investigation of changes in brain structure in patients suffering from painful TMD (p-TMD). The CT in the left lateral occipital and the FD in the left paracentral, right pars opercularis, right rostral middle frontal, left lingual, and right inferior temporal were shown to be significantly reduced in p-TMD patients when compared to NC.

Both thickness and surface area can affect brain volume estimation; therefore, the increase in volume may be mainly due to the thickening of cortical thickness or the surface area. The increases in volume may not be consistent or coordinated with the equivalent changes in surface area and thickness. Consequently, an independent parameter is better targeted by the SBM approach.

Compared with the single brain region with decreased CT in this study, Moayedi et al. found that patients with TMD had cortical thickening in multiple regions [15]. This may be due to the different disease durations of the enrolled patients, and the increase in cortical thickness may be the compensatory manifestation of the brain structure in the p-TMD patients. Additionally, earlier research had demonstrated that patients with cluster headaches had reduced cortical thickness in both the precentral and angular gyrus; however, there was no discernible relationship between the reduction and the length of the illness, suggesting the potential role of cortical structures in the pathogenesis of cluster headache [16]. Nevertheless, this study observed decreased CT only in the left lateral occipital of p-TMD individuals. Drawing from both the present research and prior investigations, it might be assumed that patients experiencing distinct forms of persistent pain might have a shared neurological and pathological foundation. Therefore, the brain structure changes may be affected by personality and pain characteristics. Thus, personality and pain traits may influence changes in brain anatomy. According to physiological principles, inflammation and cell damage can cause glial cell edema and hypertrophy, which in turn can alter the brain cortical thickness [17]. Additionally, cortical thickening may result from central functional compensation following particular neurological impairments at particular times [18]. Thus, more research is required to determine the mechanism underlying the decreased CT in p-TMD patients.

In biology and medicine, the geometrical characteristics of complex objects are frequently described using the concept of fractals. Fractal was composed of self-similar parts that vary in size and were produced through repeated iterations. They are different from geometric figures of elements in geometry and are often associated with irregular geometric objects. Fractal features can also be present in the human brain as an irregular whole [19]. Moreover, FD can be used to observe the cortical alternations during the onset and progression of numerous diseases, such as schizophrenia [20], manic-depressive [21], and Alzheimer's disease [22], since it can dynamically monitor the complex changes of brain structure and function from a fresh perspective. Reduced FD was discovered in several brain regions in p-TMD sufferers, primarily in the frontal and temporal lobes, which are associated with the intake, integration, and processing of pain information. Thus, it might be assumed that FD may assess the adaptability and efficacy of pain modulation in p-TMD patients. Consequently, we could observe the dynamic changes in FD over time in p-TMD patients following treatment and investigate its potential function in tracking the onset of pain.

We found that no noteworthy variations were observed in SD and GI between the two cohorts. SD was used to describe the distance in the sulcus between the gyrus that protruded from the brain's surface, which can reflect cortical folding. It was demonstrated that SD was considerably less throughout the majority of the cortical lobes in people with schizophrenia compared to NC [23]. The decreased SD in the temporal lobe was observed in Alzheimer’s disease, which implied disease progression from normal controls to mild cognitive impairment and then to AD [24]. As shown in some studies, the number of cortical folds was assessed using GI in a given location or the complexity of cortical folding [25-27]. Compared to NC, differences in GI in patients affected by migraine without aura may also suggest the presence of congenital and acquired abnormalities in migraine [26].

Meanwhile, trigeminal neuralgia patients exhibited decreased GI in the left superior frontal cortex and decreased SD in the bilateral superior frontal (extending to anterior cingulate) cortex [27]. Therefore, these morphological changes might contribute to understanding the underlying neurobiological mechanism of pain modulation in p-TMD patients. However, in our study, there were no statistically significant differences in SD and GI, which might be due to less prominence of neural changes or small sample sizes.

These results should still be further studied before they are used in clinical practice, even though the preliminary findings are encouraging. Firstly, the conclusion of the current analysis needs to be confirmed by a larger sample size in future studies for accurate statistics. Furthermore, the observation in this study was cross-sectional. It would be better to reveal the crucial question of whether the morphological alterations could be reversible with the right treatment. Finally, further studies should be conducted to compare how different aspects of pain, particularly pain severity, affect the structure of the brain.

## CONCLUSION

The current study revealed that p-TMD patients presented significant reductions in cortical thickness and fractal dimension, which may be associated with abnormal neural mechanisms underlying the brain's processing of emotions and pain. Furthermore, more independent morphological parameters to investigate the brain structure may be available using the SBM approach.

## Figures and Tables

**Table 1:** Demographic and clinical characteristics of the subjects.

	p-TMD	NC	*P*-value
Num(M/F)	41(18/23)	33(12/21)	0.511
Age^a^	25.00(33.00, 22.00)	25.00(26.50, 23.00)	0.785
HAMA^a^	4.00(6.00, 2.00)	3.00(6.50, 1.00)	0.450
HAMD^a^	3.00(5.00, 1.00)	2.00(4.00, 1.00)	0.305
GAD-7^a^	3.00(7.50, 0.00)	1.00(5.00, 0.00)	0.157
PHQ-9^a^	4.00(8.50, 1.00)	4.00(7.00, 2.00)	0.710
PHQ-15^a^	3.00(8.50, 0.50)	3.00(5.00, 0.00)	0.353
OBC^a^	18.00(24.50, 13.50)	15.00(26.00, 11.50)	0.439
VAS^a^	3.00(4.50, 2.00)	NA	NA

Abbreviations: HAMA: Hamilton Anxiety Scale; HAMD: Hamilton Depression Scale; GAD-7: Generalized Anxiety Disorder-7; PHQ-9: Patient Health Questionnaire-9; PHQ-15: Patient Health Questionnaire-15; OBC: Oral Behavior Checklist; VAS: Visual Analogue Score; NA: Not Available; ^a^Median (the third quartile, the first quartile);

**Table 2:** Jaw functional limitation scale of the subjects.

	p-TMD	NC	*P*-value
Chewing^a^	16.00(27.50, 3.00)	0.00(5.00, 0.00)	0.000
Movement^a^	11.00(17.00, 4.50)	0.00(2.50,0.00)	0.000
Communication^a^	5.00(13.50, 0.00)	0.00(0.00, 0.00)	0.000
General Function^a^	35.00(50.00, 16.00)	3.00(6.50, 0.00)	0.000

^a^Median (the third quartile, the first quartile);

**Table 3:** The brain regions with decreased cortical thickness in the p-TMD group compared with NC.

Anatomic Region	MNI-space			Cluster Size	*P*_uncorr_	Peak T-value
X	Y	Z
Left lateral occipital	-43	-85	-4	40	0.000	3.98

**Table 4:** The brain regions with decreased fractal dimension in the p-TMD group compared with NC.

Anatomic Region	MNI-space			Cluster Size	*P*_uncorr_	Peak T-value
X	Y	Z
Left paracentral	-11	-42	65	138	0.000	4.45
Right pars opercularis	42	19	8	206	0.000	4.39
Right rostral middle frontal	23	27	37	117	0.000	4.36
Left lingual	-16	-68	-11	45	0.000	3.98
Right inferior temporal	47	-5	-33	60	0.000	3.75

## Data Availability

All the data supporting our findings is contained within the manuscript.

## LIST OF ABBREVIATIONS

CT: Cortical Thickness
FD: Fractal Dimension
GI: Gyrification Index
SD: Sulcal Depth
GAD-7: Generalized Anxiety Disorder-7
HAMA: Hamilton Anxiety Scale
HAMD: Hamilton Depression Scale
JFLS: Jaw Functional Limitation Scale
NC: Normal Control
OBC: Oral Behavior Checklist
PHQ-9: Patient Health Questionnaire-9
PHQ-15: Patient Health Questionnaire-15
SBM: Surface Based Morphometry
TMD: Temporomandibular Disorders
TMJ: Temporomandibular Joint
VBM: Voxel Based Morphometry
VAS: Visual Analog Scale

## STANDARDS OF REPORTING

STROBE guidelines were followed.
